# Editorial: Modern Approaches to Hemophilia Management: Gene Therapy and Beyond

**DOI:** 10.3389/fmed.2022.859710

**Published:** 2022-04-13

**Authors:** Daniel Coriu, Noemi Dirzu, Alina-Andreea Zimta, Bryan C. Hambley, Xiaojing Liu, Laszlo Nemes, Gabriel Ghiaur, Johny Mahlangu, Ciprian Tomuleasa

**Affiliations:** ^1^Department of Hematology, Carol Davila University of Medicine and Pharmacy, Bucharest, Romania; ^2^Department of Hematology, Fundeni Clinical Institute, Bucharest, Romania; ^3^Medfuture Research Center for Advanced Medicine, Iuliu Hatieganu University of Medicine and Pharmacy, Cluj-Napoca, Romania; ^4^Division of Hematology/Oncology, Department of Internal Medicine, University of Cincinnati, Cincinnati, OH, United States; ^5^Department of Infectious Diseases, The First Affiliated Hospital of Xi'an Jiaotong University, Xi'an, China; ^6^National Hemophilia Center and Hemostasis Department, Medical Center of the Hungarian Defense Forces, Budapest, Hungary; ^7^Department of Oncology, Sidney Kimmel Comprehensive Cancer Center, Johns Hopkins University School of Medicine, Baltimore, MD, United States; ^8^Haemophilia Comprehensive Care Centre, Charlotte Maxeke Johannesburg Academic Hospital, University of the Witwatersrand and National Health Laboratory Service, Johannesburg, South Africa; ^9^Department of Hematology, Iuliu Hatieganu University of Medicine and Pharmacy, Cluj-Napoca, Romania; ^10^Department of Hematology, Ion Chiricuta Clinical Cancer Center, Cluj-Napoca, Romania

**Keywords:** hemophilia, modern approach, therapy, special issue, editorial

Hemophilia is a rare and life-long bleeding disorder in which the blood does not clot normally due to clotting factor deficiency. While people with hemophilia can lead near normal lives with certain precautions to prevent and treat bleeds, living with hemophilia has many challenges. Those living with hemophilia or caring for someone with hemophilia face a wide range of medical, psychological, social, and financial difficulties which is why a strong network of support is a vital part of comprehensive care. Nevertheless, notable drawbacks include occasionally insufficient hemostatic protection, the need for repeated prophylactic injections, as well as the variable risk of developing alloantibodies, which are conventionally known as inhibitors. Substantial advances have also since been achieved with the development of by-passing agents, manufactured primarily for managing bleeds in patients with high-titer inhibitors, as well as with manufacturing of RNA interference compound targeting antithrombin, or clotting factor mimetics based on humanized bispecific antibodies, as is the case of emicizumab (1). Recently, gene therapy brought the prospect of providing a definitive cure for bleeding disorders, rather than prophylactic or emergent management and is indeed clinically, socially, and economically attractive, due to the huge medical, social, and economic burden that hemophilia imposes.

The present manuscript is an editorial that outlines the articles submitted and published in the present Research Topic on hemophilia diagnosis and management. The purpose of the manuscript is to introduce the contributors and topic of the special issue.

Mahlangu presents a very comprehensive review on the recent progress in developing anti-tissue factor pathway inhibitors (anti-TFPI) therapies and concluded that data from phase I and Phase II clinical trials are promising, with two molecules being currently investigated entering phase III clinical trials. Further clinical trial evaluation are of utmost importance as severe side-effects of anti-TFPI include thrombotic events, managed by better understanding of the pharmacokinetics and proper dose-adjustment.

A coagulopathy is diagnosed in the hematology department both for hemophilia, as well as in hematological malignancies. One hematological malignancy in which coagulopathy is frequently described, with important clinical consequences is acute promyelocytic leukemia (APL). This is a clinical scenario presented by Hambley et al., that describe both the mechanism of hemorrhage as well the therapeutical options for clinicians. Also, as in APL malignant cells induce a pro-coagulant state, they describe the mechanism of thrombosis and its clinical management, as well the practical strategies to mitigate coagulopathy in this subtype of leukemia.

The genetic, as well as the epigenetic background of coagulation disorders, is complex and newly published data brings forward new and interesting aspects of these diseases. For example, non-mutational causes are described by Zimta et al. in acquired hemophilia, that looked at the genetic and transcriptomic causes of hemophilia. They hypothesized that acquired hemophilia may also be caused by either epigenetic changes or by non-coding RNAs (ncRNA). Still, this hypothesis must be either validated or invalidated by future experimental data.

No matter of coagulation disorders include “classic” hemophilia or other coagulopathies, may it be acquired hemophilia or coagulation associated with a malignancy, may it hematological or a solid malignancy, patient monitoring is the next step forward. In the 2020 guidelines of the World Federation of Hemophilia, software-based patient diagnosis and monitoring was promoted. Dirzu et al. present mobile health technology (mHealth) in a critical and comprehensive way, describing both the very obvious advantages as well as the disadvantages. Still, such applications, along with other IT-based technologies as is the case of national registries, are extremely important to properly diagnose treat and monitor patients with coagulation disorders and should be implemented in the near, if not immediate, future.

Last, but certainly not least, Yang et al. showed that next generation sequencing can be used to diagnose hereditary spherocytosis and thus identifying mutated genes can not only accurately treat diseases, but also avoid potential genetic risks and improve prenatal and postnatal care.

## Author Contributions

All authors contributed in writing the manuscript. All authors contributed to the article and approved the submitted version.

## Funding

CT was supported by five grants awarded by the Romanian National Ministry of Research, Innovation, and Digitalization: Postdoctoral Research Project PN-III-P1-1.1-PD-2019-0805, No. PD122/2020; CCCDI-UEFISCDI, Project No. PN-III-P4-ID-PCCF-2016–0112 within PNCDI III, for Young Research Teams 2020–2022 (Grant No. PN-III-P1-1.1-TE-2019-0271); PN-III-P4-ID-PCE-2020-1118 within PNCDI IV, Projects for Exploratory Medicine; Projects for Exploratory Medicine—PCE 225/2021; PN-III-CEI-BIM-PBE-2020-0016 within PNCDI I—collaboration between Romania and Belgium (Wallonia), contract number 13-BM/2020; as well as by two other international collaborative grants of the European Economic Space between Romania and Iceland 2020–2022: grant No. 19-COP-0031—HERO; and grant, Cooperation strategy for knowledge transfer, internationalization, and curricula innovation in the field of research education at the 3rd level of study—AURORA.

## Conflict of Interest

The authors declare that the research was conducted in the absence of any commercial or financial relationships that could be construed as a potential conflict of interest.

## Publisher's Note

All claims expressed in this article are solely those of the authors and do not necessarily represent those of their affiliated organizations, or those of the publisher, the editors and the reviewers. Any product that may be evaluated in this article, or claim that may be made by its manufacturer, is not guaranteed or endorsed by the publisher.
